# Nanopore Long-Read Guided Complete Genome Assembly of *Hydrogenophaga intermedia*, and Genomic Insights into 4-Aminobenzenesulfonate, *p*-Aminobenzoic Acid and Hydrogen Metabolism in the Genus *Hydrogenophaga*

**DOI:** 10.3389/fmicb.2017.01880

**Published:** 2017-10-04

**Authors:** Han M. Gan, Yin P. Lee, Christopher M. Austin

**Affiliations:** ^1^Centre for Integrative Ecology, School of Life and Environmental Sciences, Deakin University, Geelong, VIC, Australia; ^2^Genomics Facility, Tropical Medicine and Biology Platform, Monash University Malaysia, Bandar Sunway, Malaysia; ^3^School of Science, Monash University Malaysia, Bandar Sunway, Malaysia

**Keywords:** *Hydrogenophaga*, Nanopore, comparative genomics, hydrogen metabolism, phylogeny

## Abstract

We improved upon the previously reported draft genome of *Hydrogenophaga intermedia* strain PBC, a 4-aminobenzenesulfonate-degrading bacterium, by supplementing the assembly with Nanopore long reads which enabled the reconstruction of the genome as a single contig. From the complete genome, major genes responsible for the catabolism of 4-aminobenzenesulfonate in strain PBC are clustered in two distinct genomic regions. Although the catabolic genes for 4-sulfocatechol, the deaminated product of 4-aminobenzenesulfonate, are only found in *H. intermedia*, the *sad* operon responsible for the first deamination step of 4-aminobenzenesulfonate is conserved in various *Hydrogenophaga* strains. The absence of *pabB* gene in the complete genome of *H. intermedia* PBC is consistent with its *p*-aminobenzoic acid (pABA) auxotrophy but surprisingly comparative genomics analysis of 14 *Hydrogenophaga* genomes indicate that pABA auxotrophy is not an uncommon feature among members of this genus. Of even more interest, several *Hydrogenophaga* strains do not possess the genomic potential for hydrogen oxidation, calling for a revision to the taxonomic description of *Hydrogenophaga* as “hydrogen eating bacteria.”

## Introduction

The genus *Hydrogenophaga* consists of rod-shaped yellow-pigmented gram negative bacteria that are generally considered capable of using hydrogen as an energy source (Willems et al., 1989). Among all currently described species of *Hydrogenophaga*, *Hydrogenophaga intermedia* is one of the most studied species due to its distinctive ability to efficiently degrade 4-aminobenzenesulfonate (4-aminobenzenesulfonate), a recalcitrant intermediate compound in the synthesis of colorants (Dangmann et al., 1996). The catabolism of 4-aminobenzenesulfonate in *H. intermedia* S1 is by far the most well-studied among all described 4-aminobenzenesulfonate degraders (Perei et al., 2001; Wang et al., 2009; Gan et al., 2011b; Yang et al., 2012; Hayase et al., 2016). *H. intermedia* S1 was isolated from wastewater and could grow on 4-aminobenzenesulfonate as the sole carbon source in a two-species bacterial coculture with *Agrobacterium radiobacter* S2 (Dangmann et al., 1996). To date, several enzymes associated with the downstream degradation of 4-aminobenzenesulfonate in strain S1 have been identified, cloned, characterized and functionally validated (Contzen et al., 2001; Halak et al., 2006, 2007). However, the first deamination step converting 4-aminobenzenesulfonate to 4-sulfocatechol, which was presumed to be catalyzed by a dioxygenase is yet to be indentified in strain S1 (Contzen et al., 2001; Halak et al., 2006, 2007).

More than 20 years after the isolation of the S1/S2 mixed culture, a second 4-aminobenzenesulfonate-degrading two-species bacterial culture consisting of *Hydrogenophaga* sp. PBC and *Ralstonia* sp. PBA isolated from textile wastewater was reported (Gan et al., 2011b). Transposon mutagenesis of strain PBC led to the identification of the *sad* operon that is responsible for the initial deamination step of 4-aminobenzenesulfonate to 4-sulfocatechol. The deamination step was presumed to operate similarly *H. intermedia* strain S1 given its near identical 16S rRNA sequence to strain PBC (Gan et al., 2011a,b, 2012c). In addition, both *Hydrogenophaga* strains could only be grown in axenic culture with 4-aminobenzenesulfonate as the sole carbon and nitrogen source if they were supplemented with *p*-aminobenzoate and biotin (Kampfer et al., 2005; Gan et al., 2011a,b), providing insight into the syntrophic relationship between the *Hydrogenophaga* strains and their helper strains in addition to suggesting that the biosynthesis pathways for these compounds were either missing or non-functional in *H.* sp. PBC and *H. intermedia* S1 (Dangmann et al., 1996; Gan et al., 2011b).

Strain PBC was the first *Hydrogenophaga* strain to have its genome sequenced (Gan et al., 2012b). However, due to the short read length of Illumina sequencing (2 × 100 bp) at that time and the high genomic GC content of strain PBC, the assembled genome was relatively fragmented. Further, most of the genes coding for 4-aminobenzenesulfonate metabolism were located on different contigs, limiting the analysis of the genomic structure and regulation of this pathway (Hegedüs et al., 2017). Interestingly, genes coding for biosynthesis of biotin and 4-aminobenzoic acid could not be identified in the draft genome of strain PBC, which is consistent with its requirement for these supplementations for growth in axenic culture (Gan et al., 2011b; Kim and Gan, 2017). However, given that gaps exist in the draft genome, it may be possible that these gene regions were not assembled, thus emphasizing the need for a complete genome assembly to verify this observation. As more *Hydrogenophaga* genomes became available, *H.* sp. PBC was recently reclassified as *H. intermedia* PBC based on *in silico* genome–genome hybridization (Kim and Gan, 2017). Interestingly, despite the availability of various whole genome sequences for the type species of *Hydrogenophaga*, comparative genomic analyses of *Hydrogenophaga* currently are limited and most analyses have focused on a single genome (Gan et al., 2011b; Kim and Gan, 2017).

One of the defining traits of the genus *Hydrogenophaga* is the ability to grow chemorganotrophically or chemolithoautotrophically by oxidizing hydrogen as an energy source (Willems et al., 1989). A [NiFe]-membrane-bound hydrogenase has been isolated and purified from *H.* sp. AH-42 providing the first biochemical characterization of hydrogenase and molecular insight into the genetic components responsible for hydrogen oxidation in the genus *Hydrogenophaga* (Yoon et al., 2008, 2009). However, exceptions to the defining feature of the genus, e.g., inability to oxidize hydrogen under standardized culture condition, have been reported in two *Hydrogenophaga* type strains namely *H. intermedia* S1^T^ and *H. atypica* DSM 15342^T^ (Contzen et al., 2000; Kampfer et al., 2005), naturally inviting a comprehensive genomic survey of hydrogen-oxidizing genes in all sequenced members of the genus *Hydrogenophaga*.

To improve the genome assembly of *H. intermedia* PBC, we used the Nanopore MinION portable long-read sequencer to generate long sequencing reads coupled with a hybrid assembly using the Illumina dataset generated previously by Gan et al. (2012b). We showed that the incorporation of the Nanopore long reads enabled the successful assembly of *H. intermedia* PBC genome into a single contig. Further, we also provided an updated whole genome-based phylogeny of the family Comamonadaceae in addition to performing comparative genomics analysis of the genus *Hydrogenophaga* for the first time focusing on the prevalence of genomic potential for hydrogen oxidation, pABA synthesis and 4-aminobenzenesulfonate biodegradation.

## Materials and Methods

### Illumina and Nanopore Whole Genome Sequencing

Illumina-based whole genome sequencing and assembly of strain PBC has been previously described (Gan et al., 2012b). To generate sequencing data using the MinION device, the gDNA of strain PBC was extracted from a 5-day old culture on nutrient agar using a slightly modified SDS-lysis method (Sokolov, 2000). Five micrograms of gDNA was subsequently used for library construction using the Nanopore sequencing kit SQK-NSK007 (Oxford Nanopore Technologies, Oxford, United Kingdom) according to the manufacturer’s instructions. The library was subsequently loaded onto an R9 MINION flowcell (Oxford Nanopore Technologies, Oxford, United Kingdom) and run for 48 h. 2D-basecalling was performed on the cloud-based Metrichor. Fast5 to fasta conversion used Nanocall (David et al., 2016).

### Hybrid Genome Assembly and Genome Annotation

SPAdes version 3.8.1 (Antipov et al., 2016) was used for an initial hybrid genome assembly incorporating both Illumina and Nanopore reads. Contigs longer than 1,000 bp were then selected for *in silico* scaffolding and gap-closing using npScarf (Cao et al., 2016). The assembled genome was annotated using the NCBI Prokaryotic annotation pipeline (Tatusova et al., 2016) and manually curated to include annotations for the *pcaH2, pcaG2*, *pcaB2* and 4SLH genes coding for protocatechuate 3,4-dioxygenase alpha subunit type II, protocatechuate 3,4-dioxygenase beta subunit type II, 3-carboxymuconate cycloisomerase type II and 4-sulfomuconolactoe hydrolase, respectively, that were missed by the default annotation setting. The identification and annotation of prophage sequences were performed using the PHASTER web-server (Arndt et al., 2016). Visualization of the complete genome of strain PBC and genomic comparisons with public available *Hydrogenophaga* genomes (**Table 1**) were performed using Blast Ring Image Generator (blastN *e*-value setting of 1e-10) (Alikhan et al., 2011). Mapping of Nanopore reads and Illumina contigs was performed using Minimap2^1^ and subsequently visualized in Integrative Genomics Viewer version 3 (Thorvaldsdóttir et al., 2013). Generation of Illumina contigs was done by assembling Illumina-only reads using SPAdes version 3.8.1 and filtering for contigs with coverage and length of more than 5× and 300 bp, respectively (Antipov et al., 2016).

**Table 1:** *Hydrogenophaga* strains used for comparative genomics analysis.

Organism	Accession number	Isolation source	Reference
*Hydrogenophaga intermedia* PBC	CP017311	Textile waste water	This study
*Hydrogenophaga intermedia* S1 (DSM 5680^T^)	CCAE01	Waste water with 4-aminobenzenesulfonate as sole source of carbon	Kim and Gan, 2017
*Hydrogenophaga* sp. Root209	LMIE01	Root of *Arabidopsis thaliana*	Bai et al., 2015
*Hydrogenophaga pseudoflava* NBRC 102511^T^	BCWQ01	River water	unpublished
*Hydrogenophaga palleronii* NBRC 102513^T^	BCTJ01	Water enriched for hydrogen bacteria in an atmosphere containing 6% oxygen	unpublished
*Hydrogenophaga taeniospiralis* NBRC 102512^T^	BCWR01	Soil	unpublished
*Hydrogenophaga flava* NBRC 102514^T^	BCTF01	Mud from ditch	unpublished
*Hydrogenophaga* sp. RAC07	CP016449	Algal phycosphere	Fixen et al., 2016
*Hydrogenophaga* sp. LPB0072	LVWD01	Pacific oyster (*Crassostrea gigas*)	Unpublished
*Hydrogenophaga* sp. PML113	MIYM01	Contaminated water	Unpublished
*Hydrogenophaga* sp. IBVHS2	NFUT01	Unknown	Unpublished
*Hydrogenophaga* sp. IBVHS1	NFUU01	Unknown	Unpublished
*Hydrogenophaga* sp. A37	MUNZ01	Fjaler soil	Unpublished
*Hydrogenophaga* sp. H7	MCIC01	Coal mine	Unpublished


### *In Silico* Genome–Genome Hybridization

Pair-wise average nucleotide identity of strain PBC against the publicly available genome sequences of *Hydrogenophaga* species were performed using JSpecies V1.2 (ANIm setting) (Richter et al., 2016). Strains exhibiting pair-wise ANI of more than 95% were considered as members of the same genospecies. A heatmap was plotted in R version 3 using the pheatmap library package.

### Whole Genome Phylogeny

Whole proteome was predicted using Prodigal (-p meta setting) and piped into PhyloPhlan for the identification of conserved proteins (Hyatt et al., 2010; Segata et al., 2013). The concatenated alignment generated by PhyloPhlAN was subsequently trimmed using trimAl version 1.9 (-automated1 setting) and used to construct a maximum likelihood tree using IqTree version 5.15 with 1000 ultrafast bootstraps (Capella-Gutiérrez et al., 2009; Nguyen et al., 2015). Visualization and annotation of trees was performed with MEGA6 (Tamura et al., 2013).

### Comparative Genomics Analysis

HMMsearch version 3.1b was used for the identification of proteins of interest utilizing either the PFAM (-E 1e^-5^ setting) or TIGRFAM (-cut_nc or –E 1e^-5^ setting) HMM profiles (Haft et al., 2003; Eddy, 2011). Protein alignment, trimming, phylogenetic tree construction and visualization were performed using MAFFT-linsi, TrimAl, IqTree and FigTree, respectively (Capella-Gutiérrez et al., 2009; Katoh and Standley, 2013; Nguyen et al., 2015). Visualization of gene neighborhood was conducted using EasyFig (blastN setting) (Sullivan et al., 2011).

## Results and Discussion

### MinION Output and Hybrid Genome Assembly

**FIGURE 1:**
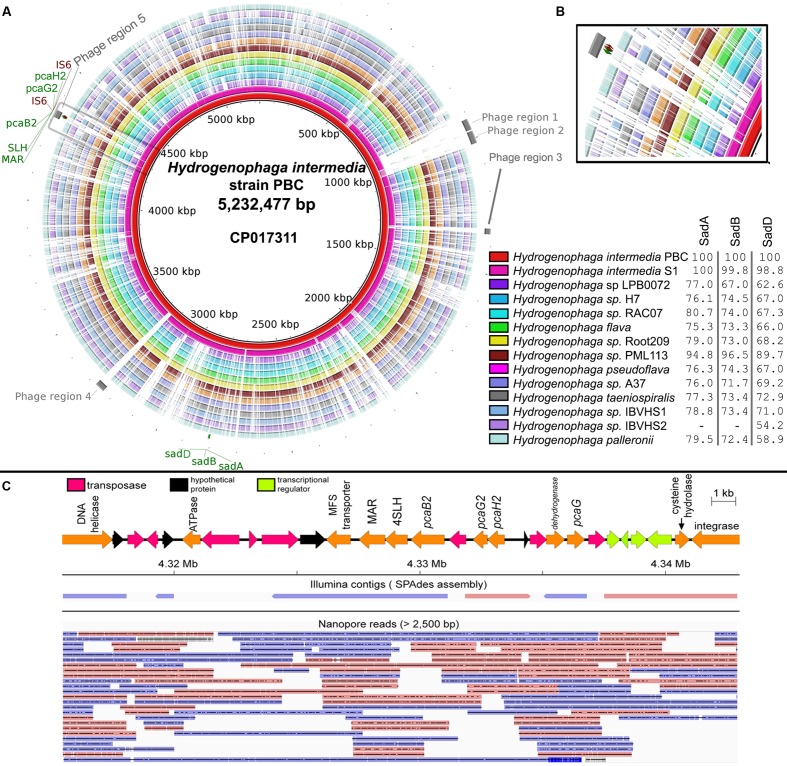
**(A)** Circular genome visualization of *Hydrogenophaga intermedia* PBC and genomic comparison with 13 additional *Hydrogenophaga* strains. Colored regions indicate region with nucleotide similarity (*E*-value < 1e-10) and percentage values next to figure legend indicate protein identity to SadA (WP_009515254.1), SadB (WP_009515256.1), and SadD (WP_009515258.1) of *H. intermedia* PBC that are involved in the deamination of 4-aminobenzenesulfonate (Gan et al., 2012c). Gray areas located at the most external ring indicate putative prophage regions. Maroon labels indicate genes coding for transposase element. *pcaG2*, protocatechuate dioxygenase type II alpha subunit; *pcaH2*, protocatechuate dioxygenase type II beta subunit; SLH, 4-sulfomuconolactone hydrolase; MAR, maleylacetate reductase; *sadA*, 4-aminobenzenesulfonate 3,4-dioxygenase; *sadB*, glutamine synthetase; *sadC*, ferredoxin; IS6, transposase. **(B)** Genomic regions containing genes associated with 4-sulfocatechol biodegradation (gray box). **(C)** Mapping of Illumina contigs and Nanopore long reads onto the genomic region containing the genes involved in 4-sulfocatechol metabolism. Blue and red arrows indicate forward and reverse strands respectively.

A total of 90,491 1D and 51,630 2D reads were generated from a full 48-h MinION run. After filtering for reads longer than 2,500 bp, 14,404 1D and 12, 959 2D reads were retained for genome assembly. The final data output used for assembly was 173 megabases representing approximately 33× genome coverage of strain PBC. The average read length was 6,350 bp and the longest read generated was 126,959 bp. Initial Spades hybrid assembly using both Illumina and MinION filtered reads generated 5 scaffolds (>10x coverage, >1,000 bp) which were subsequently scaffolded and gap-closed into a single contig using Npscarf. The complete genome of strain PBC is 5,232,477 bp in length with a GC content of 68.4%. Based on NCBI annotation pipeline, the complete genome consists of 4,904 protein-coding genes, 43 tRNA and single-copy 5S, 16S, and 23S rRNA genes. Comparisons of strain PBC and other *Hydrogenophaga* species revealed two genomic regions (**Figure 1A**: Regions 3 and 4) uniquely present in strain PBC, that were subsequently identified as prophage in origin by Phaster. Both phage regions were incomplete and only code for two to three major phage components. Phage components 1 and 2 on the contrary are complete, uniquely shared by both *H. intermedia* strains (**Figure 1A**) and have the highest number of homologous proteins to Enterobacter phage Arya (NCBI Reference Sequence: NC_0310480) isolated from a termite gut.

### New Genomic Insight into 4-Aminobenzenesulfonate Biodegradation in *Hydrogenophaga*

Genes responsible for the catabolism of 4-aminobenzenesulfonate in strain PBC are clustered in two distinct genomic regions. Interestingly, the *sad* operon appears to be fairly conserved in all currently reported *Hydrogenophaga* genomes, suggesting that the ability to deaminate 4-aminobenzenesulfoate may be a defining metabolic trait of *Hydrogenophaga* strains. However, given the diverse isolation sources of *Hydrogenophaga* strains (**Table 1**) and their presumably limited exposure to xenobiotic compounds, it is likely that the original substrate of the 4-aminobenzenesulfonate dioxygenase is a naturally occurring compound with close structural homology to 4-aminobenzenesulfonate.

In contradistinction, the genomic region containing *pcaH2G2, pcaB2* and 4-SLH involved in the conversion of 4-sulfocatechol to maleylacetate is conserved only in the *Hydrogenophaga intermedia* strains (**Figure 1B**). In addition, it is also worth noting that this region was identified as a phage region and flanked by genes coding for IS6 transposable elements (**Figure 1C**), an indication of the mobility of the *pcaH2G2*, *pcaB2* and 4-SLH gene cluster presumably through phage transduction (Hickman et al., 2010). As expected, the presence of multiple transposase genes in this genomic region led to a fragmented initial Illumina read assembly (**Figure 1C**) due to the inability of short reads to resolve the repetitive regions (Phillippy, 2017). This shortcoming was overcome by integrating Nanopore long reads into the assembly as evidenced by their ability to span repeats in the genome (**Figure 1C**).

It is tempting to speculate that the acquisition of this gene cluster may be a key factor leading to complete 4-aminobenzenesulfonate biodegradation in *Hydrogenophaga*. Recently, the complete genome of *Novosphingobium resinovorum* SA1, another well-studied 4-aminobenzenesulfonate degrader, has been reported and comparison of this genomic region and that of the homologous region in *Novosphingobium resinovorum* SA1, revealed a comparable gene organization, e.g., *pcaH2-pcaG2-tnp-pcaB2*-4SLH-MAR in *H. intermedia* PBC (**Figure 1C**) and *pcaB2* (*scaA*)-4SLH (*scaB*)-MAR(*scaC*)-oxidoreductase-*pcaH2*(*scaE*)-*pcaG2*(*scaF*) in *Novosphingobium resinovorum* SA1 (Hegedüs et al., 2017), suggesting recently shared ancestry of the currently known 4-sulfocatechol biodegradation pathway and possibly inter-class (alphaproteobacteria-betaproteobacteria) horizontal gene transfer of this gene cluster.

### *H. pseudoflava* and *H. flava* Belong to Two Genospecies Despite Their High Similarity in Genotypic and Protein Electrophoretic Profiles

**FIGURE 2:**
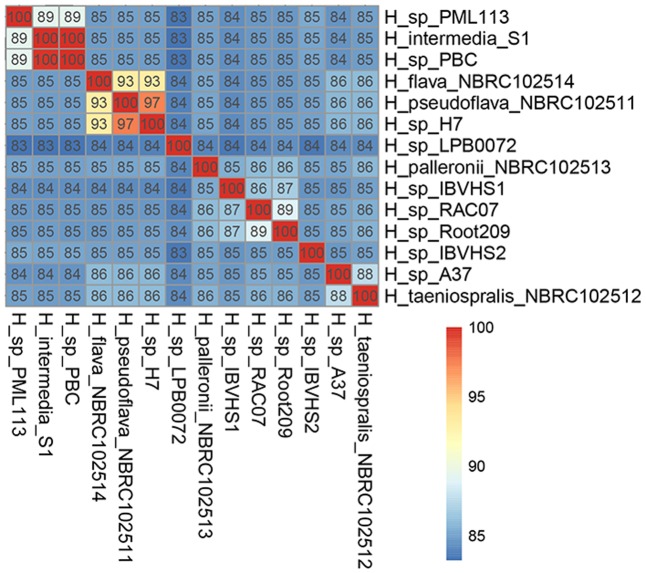
Pairwise average nucleotide similarity of *Hydrogenophaga* strains.

*Hydrogenophaga flava* was initially designated as the type species of the genus *Hydrogenophaga* but due to its slow and unreliable growth, *H. pseudoflava* has been proposed for use as an alternative reference taxon for the genus as it exhibits similar genotypic and protein electrophoretic profiles to that of *H. flava* (Willems et al., 1989). *In silico* genome-genome hybridization indicates that although *H. flava* and *H. pseudoflava* are closely related to one another, they are clearly two distinct genospecies with a pairwise ANIm of 93% (**Figure 2**). A more in-depth analysis of the *H. flava* genome may be useful to identify its additional nutritional requirements for optimal and consistent growth on culture medium. Such a genomic approach has been successfully demonstrated in *Clostridium tyrobutyricum* and other Clostridia associated with butyric acid fermentation leading to the identification of key vitamins and several amino acids essential for growth (Storari et al., 2016). Furthermore, *in silico* genome-genome hybridization analysis also indicates that *H.* sp. H7, which was previously isolated from a coal mine in China (**Table 1**), could be reclassified to the species *H. pseudoflava* given its high pairwise ANIm (97%) to *H. pseudoflava* NBRC 102511^T^ (**Figure 2**).

### Genome-Based Phylogeny of the Family Comamonadaceae Supports *Hydrogenophaga* as a Monophyletic Group but Indicates Taxonomic Incongruence in the Genera *Acidovorax* and *Comamonas*

**FIGURE 3:**
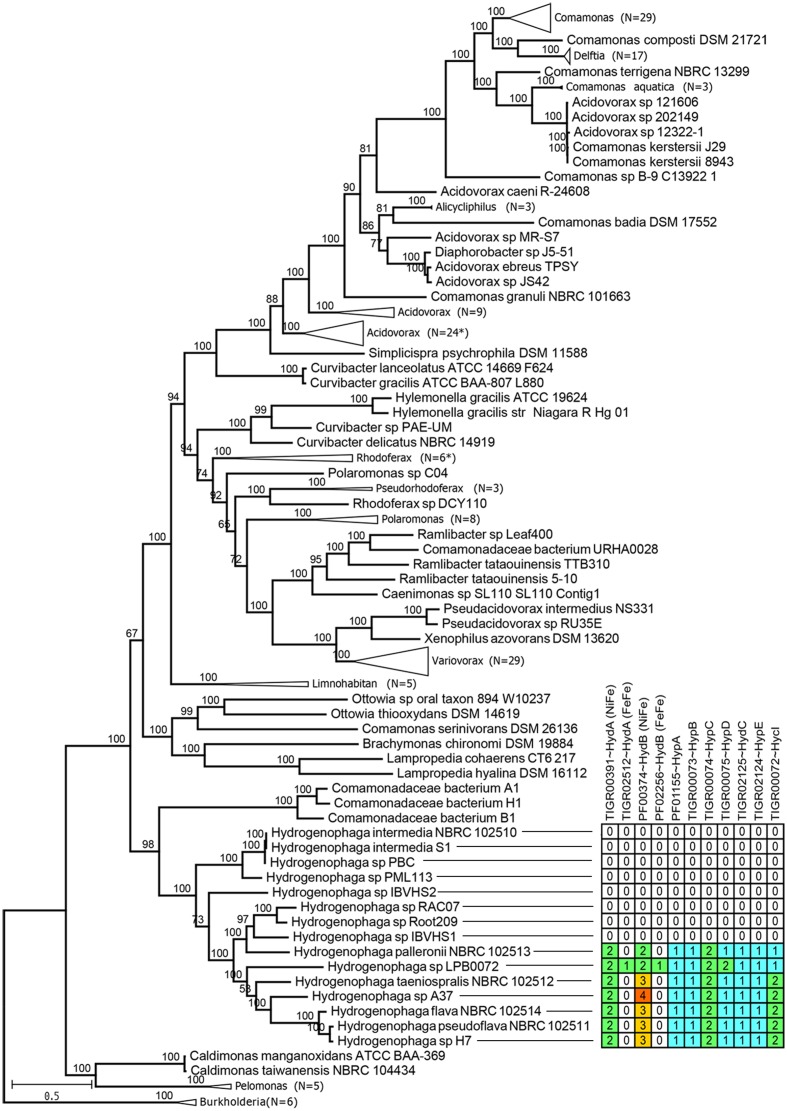
Maximum likelihood phylogenomic tree of the family Comamonadaceae. The tree was rooted with members of the genus *Burkholderia* belonging to the family Burkholderiaceae, as the outgroup. Numbers in brackets indicate the number of branches collapsed. Asterisks indicate the presence of one member with inconsistent taxonomic labeling, e.g., different genus name within the collapsed branch. Numbers above branches indicate ultra-fast bootstrap support values. Table next to the *Hydrogenophaga* clade show the number of proteins showing significant hits to HMM profiles associated with bacterial hydrogenase system.

Maximum likelihood tree based on 400 universal conserved proteins provides maximal support for the monophyletic clustering of all currently sequenced *Hydrogenophaga* strains with *H. intermedia* strains and *H.* sp. PML 113 being basal to the rest of *Hydrogenophaga* (**Figure 3**). The genus *Hydrogenophaga* clade shares a sister relationship with a group of highly alkaliphilic hydrogen-utilizing Comamonadacea bacteria isolated from an alkaline serpentinizing springs at The Cedars, California (Suzuki et al., 2014). Beyond the genus *Hydrogenophaga*, however, considerable taxonomic incongruence was observed in the genera *Acidovorax* and *Comamonas*. Some members of the *Acidovorax*, such as *Acidovorax* sp. strains 121606, 202149 and 12322-1, could be reclassified as members of the genus *Comamonas* based on their phylogenetic affinities. Within the genus *Comamonas*, *Comamonas badia* DSM 17552^T^ (Suzuki et al., 2014) is peculiar as it does not cluster with a majority of other *Comamonas*, but instead forms a sister group with members of the genus *Alicycliphilus*, albeit with moderate bootstrap support. In addition, *Comamonas granuli* NBRC 101663^T^ appears to be considerably divergent from other *Comamonas* species given its basal position in the clade containing all currently sequenced *Comamonas* strains (Suzuki et al., 2014). A more in-depth taxonomic investigation based on the percentage of conserved proteins (POCP) to specifically re-define the genus boundary between *Comamonas* and *Acidovorax* should be undertaken (Qin et al., 2014).

### Genomic Potential for Hydrogen Oxidation Is Restricted to a Few Members of *Hydrogenophaga*

The genus *Hydrogenophaga* was originally proposed by Willems et al. (1989) to group “yellow-pigmented hydrogen-oxidizing [*Pseudomonas*] species belonging to the acidovorans rRNA complex.” However, by identifying currently known HMM profiles related to hydrogenase system, genomic potential for hydrogen oxidation was only identified in 7 *Hydrogenophaga* strains of which 6 strains are closely related as shown by their monophyletic clustering in the phylogenomic tree (**Figure 3**). The 7 *Hydrogenophaga* strains include the original strains that were initially used to describe the genus *Hydrogenophaga*, such as *H. palleronii, H. taeniospralis, H. flava and H. pseudoflava*, corroborating their previously demonstrated *in vitro* hydrogen oxidizing ability (Willems et al., 1989; Contzen et al., 2000). The reported inability of *H. intermedia* S1^T^ and *H. atypica* DSM 15342^T^ to oxidize hydrogen *in vitro* (Contzen et al., 2000; Kampfer et al., 2005) correlates with the absence of key genes associated with hydrogen metabolism (**Figure 3**). However, the absence of genomic potential for hydrogen catabolism in nearly half of the currently sequenced *Hydrogenophaga* strains is unexpected and indicates that the description genus of *Hydrogenophaga* as “hydrogen eating bacteria” is not sustainable and warrants revision.

### *p*-Aminobenzoic Acid Auxotrophy within *Hydrogenophaga*

**FIGURE 4:**
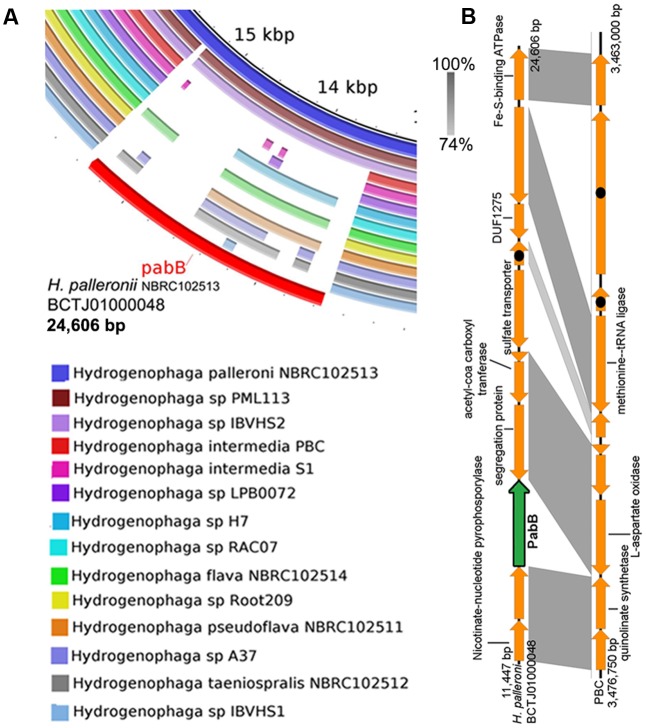
Prevalence of *pabB* among Hydrogenophaga strains based on genomic and phylogenetic analyses. **(A)** Homology analysis against the *pabB*-containing genomic region of *Hydrogenophaga palleronii* **(B)** Easyfig visualization of homologous genomic regions containing *pabB* between *H. intermedia* PBC and *H. palleronii*. Shaded regions indicate regions with *E*-value score of less than 1e^-5^ with the percentage of nucleotide identity expressed along a gray scale. Hypothetical proteins are indicated by arrows containing black circles.

Mapping of *Hydrogenophaga* genome sequences to the contig of *H. palleronii* NBRC102513 (BCTJ01000048, 24 kbp) containing *pabB* revealed that only 8 out of 14 genomes exhibit nucleotide similarity (>40% identity and *E*-value < 1E^-5^) to the *pabB* gene region (**Figure 4A**). Of the eight genomes, only partial *pabB* hits were observed for strains H7, NBRC102514, Root209, NBRC102511 and A37, hinting divergence at the nucleotide level. Interestingly, only two proteins belonging to *H. palleroni* and *H.* sp. *PML113* achieved an HMMscore that is above the domain noise cutoff score for bona fide PabB (TIGR00553) (**Supplementary Figure S1**). On the contrary, strain IBVHS2 despite exhibiting nucleotide similarity to the *pabB* gene region, does not code for protein with HMMscore above the TIGR00553 noise cutoff. However, it is worth noting that multiple proteins with HMMscore slightly below the TIGR00553 noise cutoff could be identified from the annotated *Hydrogenophaga* genomes (**Supplementary Figure S1**). These proteins appear to be more closely related to PabB than TrpE as evidenced by their extremely low HMMscore to TrpE (TIGR00564) in comparison with other putative TrpE proteins and their phylogenetic affiliation to the PabB clade (**Supplementary Figure S2** and **Data Sheet 1**). TrpE is involved in the synthesis of anthranilate from chorismate and ammonia. Functional characterization of these divergent PabB homologs will be useful to explore their role in pABA synthesis as of them belong to *Hydrogenophaga pseudoflava* that has been shown to grow on minimal medium without pABA supplementation (Povolo et al., 2013).

The absence *pabB* in *H. intermedia* strains PBC and S1^T^ is consistent with their previously reported reliance on either external nutrient supplementation or helper strain for pABA (Dangmann et al., 1996; Gan et al., 2011b; Kim and Gan, 2017). A recent genomic analysis of *Hydrogenophaga intermedia* PBC helper strain, *Ralstonia* sp. PBA, showed the presence of two PabB proteins of which one of them was fused to PabC (Gan et al., 2012a; Kim and Gan, 2017), potentially enabling the overproduction of pABA to sustain the growth of both strains when they were co-cultured in vitamin-free minimal medium containing 4-aminobenzenesulfonate as the sole carbon and nitrogen source. A closer comparison of the genomic region containing genes spatially associated with *pabB* in *H. intermedia* PBC and *H. palleroni* NBRC102513 suggests that *pabB* may be lost through gene deletion (**Figure 4B**). Surprisingly, strains LPB0072, RAC07, Root209 and IBVHS1 appear to also lack the genomic potential for pABA synthesis. A future study investigating the ability of these strains to grow on defined medium without pABA supplementation will be necessary to verify their predicted pABA auxotrophy as most *Hydrogenophaga* strains were isolated on nutrient rich medium, which will contain traced amount of pABA. For example, strains Root209 and RAC07 were maintained on tryptic soy agar and minimal medium with yeast extract supplementation, respectively (Bai et al., 2015; Fixen et al., 2016).

## Data Access

The complete genome of *Hydrogenophaga intermedia* PBC has been deposited under the accession number CP017311. Raw data for both Illumina and Nanopore data have been placed under the SRA under the SRA project ID SRP092076.

## Author Contributions

HG performed gDNA extraction, and bioinformatics analysis and drafted the manuscript. YL performed library preparation and MINION sequencing. CA supervised the study.

## Conflict of Interest Statement

The authors declare that the research was conducted in the absence of any commercial or financial relationships that could be construed as a potential conflict of interest.

## Footnotes

1 https://github.com/lh3/minimap2

## Supplementary Material

**FIGURE S1 |** Sequence scores of Hydrogenophaga proteins with significant hits (*E*-value < 10^-5^) to TIGR00564 or TIGR00553. Dotted lines represent the TIGRFAM model noise cutoff bit score thresholds.

**FIGURE S2 |** Maximum likelihood tree of TrpE and PabB. Node values indicate ultrafast bootstrap support values. Red branches indicate proteins with high sequence scores to TIGR00564 corresponding to bona fide TrpE. Blue and green branches indicate proteins with low scores to TIGR00564 but high scores to TIGR00553 (bona fide PabB). In addition, proteins associated with blue branches exhibit scores that are either higher than or very close to the noise cutoff score of TIGR00553.

**DATA SHEET 1 |** Hydrogenophaga protein sequences used for the construction of maximum likelihood tree.

## References

[B1] Alikhan N. F. Petty N. K. Ben Zakour N. L. Beatson S. A. (2011). BLAST ring image generator (BRIG): simple prokaryote genome comparisons. *BMC Genomics* 12:402 10.1186/1471-2164-12-402 PMC3163573 21824423

[B2] Antipov D. Korobeynikov A. McLean J. S. Pevzner P. A. (2016). hybridSPAdes: an algorithm for hybrid assembly of short and long reads. *Bioinformatics* 32 1009–1015. 10.1093/bioinformatics/btv688 26589280 PMC4907386

[B3] Arndt D. Grant J. R. Marcu A. Sajed T. Pon A. Liang Y. (2016). PHASTER: a better, faster version of the PHAST phage search tool. *Nucleic Acids Res.* 44 W16–W21. 10.1093/nar/gkw387 27141966 PMC4987931

[B4] Bai Y. Muller D. B. Srinivas G. Garrido-Oter R. Potthoff E. Rott M. (2015). Functional overlap of the *Arabidopsis* leaf and root microbiota. *Nature* 528 364–369. 10.1038/nature16192 26633631

[B5] Cao M. D. Ganesamoorthy D. Elliott A. G. Zhang H. Cooper M. A. Coin L. J. M. (2016). Streaming algorithms for identification of pathogens and antibiotic resistance potential from real-time MinIONTM sequencing. *Gigascience* 5 32 10.1186/s13742-016-0137-2 PMC4960868 27457073

[B6] Capella-Gutiérrez S. Silla-Martínez J. M. Gabaldón T. (2009). trimAl: a tool for automated alignment trimming in large-scale phylogenetic analyses. *Bioinformatics* 25 1972–1973. 10.1093/bioinformatics/btp348 19505945 PMC2712344

[B7] Contzen M. Burger S. Stolz A. (2001). Cloning of the genes for a 4-sulphocatechol-oxidizing protocatechuate 3,4-dioxygenase from *Hydrogenophaga intermedia* S1 and identification of the amino acid residues responsible for the ability to convert 4-sulphocatechol. *Mol. Microbiol.* 41 199–205. 10.1046/j.1365-2958.2001.02505.x 11454212

[B8] Contzen M. Moore E. R. Blumel S. Stolz A. Kampfer P. (2000). *Hydrogenophaga intermedia* sp. nov., a 4-aminobenzenesulfonate degrading organism. *Syst. Appl. Microbiol.* 23 487–493. 10.1016/S0723-2020(00)80022-3 11249018

[B9] Dangmann E. Stolz A. Kuhm A. E. Hammer A. Feigel B. Noisommit-Rizzi N. (1996). Degradation of 4-aminobenzenesulfonate by a two-species bacterial coculture. Physiological interactions between *Hydrogenophaga palleronii* S1 and *Agrobacterium radiobacter* S2. *Biodegradation* 7 223–229. 10.1007/BF00058181 8782393

[B10] David M. Dursi L. J. Yao D. Boutros P. C. Simpson J. T. (2016). Nanocall: an open source basecaller for oxford nanopore sequencing data. *Bioinformatics* 33 49–55. 10.1093/bioinformatics/btw569 27614348 PMC5408768

[B11] Eddy S. R. (2011). Accelerated profile HMM searches. *PLOS Comput Biol.* 7:e1002195 10.1371/journal.pcbi.1002195 PMC3197634 22039361

[B12] Fixen K. R. Starkenburg S. R. Hovde B. T. Johnson S. L. Deodato C. R. Daligault H. E. (2016). Genome sequences of eight bacterial species found in coculture with the haptophyte *Chrysochromulina tobin*. *Genome Announc.* 4:e01162–16 10.1128/genomeA.01162-16 PMC5095461 27811091

[B13] Gan H. M. Chew T. H. Tay Y.-L. Lye S. F. Yahya A. (2012a). Genome sequence of *Ralstonia* sp. strain PBA, a bacterium involved in the biodegradation of 4-aminobenzenesulfonate. *J. Bacteriol.* 194 5139–5140. 10.1128/jb.01165-12 22933765 PMC3430363

[B14] Gan H. M. Chew T. H. Tay Y. L. Lye S. F. Yahya A. (2012b). Genome sequence of *Hydrogenophaga* sp. strain PBC, a 4-aminobenzenesulfonate-degrading bacterium. *J. Bacteriol.* 194 4759–4760. 10.1128/JB.00990-12 22887664 PMC3415511

[B15] Gan H. M. Ibrahim Z. Shahir S. Yahya A. (2011a). Identification of genes involved in the 4-aminobenzenesulfonate degradation pathway of *Hydrogenophaga* sp. PBC via transposon mutagenesis. *FEMS Microbiol. Lett.* 318 108–114. 10.1111/j.1574-6968.2011.02245.x 21323982

[B16] Gan H. M. Shahir S. Ibrahim Z. Yahya A. (2011b). Biodegradation of 4-aminobenzenesulfonate by *Ralstonia* sp. PBA and *Hydrogenophaga* sp. PBC isolated from textile wastewater treatment plant. *Chemosphere* 82 507–513. 10.1016/j.chemosphere.2010.10.094 21094980

[B17] Gan H. M. Shahir S. Yahya A. (2012c). Cloning and functional analysis of the genes coding for 4-aminobenzenesulfonate 3,4-dioxygenase from *Hydrogenophaga* sp. PBC. *Microbiology* 158(Pt 8), 1933–1941. 10.1099/mic.0.059550-0 22609751

[B18] Haft D. H. Selengut J. D. White O. (2003). The TIGRFAMs database of protein families. *Nucleic Acids Res.* 31 371–373. 10.1093/nar/gkg128 12520025 PMC165575

[B19] Halak S. Basta T. Burger S. Contzen M. Stolz A. (2006). Characterization of the genes encoding the 3-carboxy-*cis,cis*-muconate-lactonizing enzymes from the 4-sulfocatechol degradative pathways of *Hydrogenophaga intermedia* S1 and *Agrobacterium radiobacter* S2. *Microbiology* 152(Pt 11), 3207–3216. 10.1099/mic.0.29136-0 17074892

[B20] Halak S. Basta T. Burger S. Contzen M. Wray V. Pieper D. H. (2007). 4-Sulfomuconolactone hydrolases from *Hydrogenophaga intermedia* S1 and *Agrobacterium radiobacter* S2. *J. Bacteriol.* 189 6998–7006. 10.1128/JB.00611-07 17660282 PMC2045233

[B21] Hayase N. Fujikawa Y. Nakagawa K. Ushio K. (2016). Isolation and characterization of *Bradyrhizobium* sp. 224 capable of degrading sulfanilic acid. *Biosci. Biotechnol. Biochem.* 80 1663–1665. 10.1080/09168451.2016.1176521 27108596

[B22] Hegedüs B. Kós P. B. Bálint B. Maróti G. Gan H. M. Perei K. (2017). Complete genome sequence of *Novosphingobium resinovorum* SA1, a versatile xenobiotic-degrading bacterium capable of utilizing sulfanilic acid. *J. Biotechnol.* 241 76–80. 10.1016/j.jbiotec.2016.11.013 27851894

[B23] Hickman A. B. Chandler M. Dyda F. (2010). Integrating prokaryotes and eukaryotes: DNA transposases in light of structure. *Crit. Rev. Biochem. Mol. Biol.* 45 50–69. 10.3109/10409230903505596 20067338 PMC3107681

[B24] Hyatt D. Chen G. L. Locascio P. F. Land M. L. Larimer F. W. Hauser L. J. (2010). Prodigal: prokaryotic gene recognition and translation initiation site identification. *BMC Bioinformatics* 11:119 10.1186/1471-2105-11-119 PMC2848648 20211023

[B25] Kampfer P. Schulze R. Jackel U. Malik K. A. Amann R. Spring S. (2005). *Hydrogenophaga defluvii* sp. nov. and *Hydrogenophaga atypica* sp. nov., isolated from activated sludge. *Int. J. Syst. Evol. Microbiol.* 55(Pt 1), 341–344. 15653898 10.1099/ijs.0.03041-0

[B26] Katoh K. Standley D. M. (2013). MAFFT multiple sequence alignment software version 7: improvements in performance and usability. *Mol. Biol. Evol.* 30 772–780. 10.1093/molbev/mst010 23329690 PMC3603318

[B27] Kim K. Gan H. M. (2017). A glimpse into the genetic basis of symbiosis between *Hydrogenophaga* and their helper strains in the biodegradation of 4-aminobenzenesulfonate. *J. Genomics* 5 77–82. 10.7150/jgen.20216 28775791 PMC5535693

[B28] Nguyen L.-T. Schmidt H. A. von Haeseler A. Minh B. Q. (2015). IQ-TREE: a fast and effective stochastic algorithm for estimating maximum-likelihood phylogenies. *Mol. Biol. Evol.* 32 268–274. 10.1093/molbev/msu300 25371430 PMC4271533

[B29] Perei K. Rákhely G. Kiss I. Polyák B. Kovács K. L. (2001). Biodegradation of sulfanilic acid by *Pseudomonas paucimobilis*. *Appl. Microbiol. Biotechnol.* 55 101–107. 10.1007/s002530000474 11234949

[B30] Phillippy A. M. (2017). New advances in sequence assembly. *Genome Res.* 27 xi–xiii 10.1101/gr.223057.117 PMC5411783 28461322

[B31] Povolo S. Romanelli M. G. Basaglia M. Ilieva V. I. Corti A. Morelli A. (2013). Polyhydroxyalkanoate biosynthesis by *Hydrogenophaga pseudoflava* DSM1034 from structurally unrelated carbon sources. *New Biotechnol.* 30 629–634. 10.1016/j.nbt.2012.11.019 23201074

[B32] Qin Q.-L. Xie B.-B. Zhang X.-Y. Chen X.-L. Zhou B.-C. Zhou J. (2014). A proposed genus boundary for the prokaryotes based on genomic insights. *J. Bacteriol.* 196 2210–2215. 10.1128/jb.01688-14 24706738 PMC4054180

[B33] Richter M. Rossello-Mora R. Oliver Glockner F. Peplies J. (2016). JSpeciesWS: a web server for prokaryotic species circumscription based on pairwise genome comparison. *Bioinformatics* 32 929–931. 10.1093/bioinformatics/btv681 26576653 PMC5939971

[B34] Segata N. Börnigen D. Morgan X. C. Huttenhower C. (2013). PhyloPhlAn is a new method for improved phylogenetic and taxonomic placement of microbes. *Nat. Commun.* 4:2304 10.1038/ncomms3304 PMC3760377 23942190

[B35] Sokolov E. P. (2000). An improved method for DNA isolation from mucopolysaccharide-rich molluscan tissues. *J. Molluscan Stud.* 66 573–575. 10.1093/mollus/66.4.573

[B36] Storari M. Kulli S. Wüthrich D. Bruggmann R. Berthoud H. Arias-Roth E. (2016). Genomic approach to studying nutritional requirements of *Clostridium tyrobutyricum* and other Clostridia causing late blowing defects. *Food Microbiol.* 59 213–223. 10.1016/j.fm.2016.05.013 27375262

[B37] Sullivan M. J. Petty N. K. Beatson S. A. (2011). Easyfig: a genome comparison visualizer. *Bioinformatics* 27 1009–1010. 10.1093/bioinformatics/btr039 21278367 PMC3065679

[B38] Suzuki S. Kuenen J. G. Schipper K. van der Velde S. Ishii S. Wu A. (2014). Physiological and genomic features of highly alkaliphilic hydrogen-utilizing *Betaproteobacteria* from a continental serpentinizing site. *Nat. Commun.* 5:3900 10.1038/ncomms4900 PMC4050266 24845058

[B39] Tamura K. Stecher G. Peterson D. Filipski A. Kumar S. (2013). MEGA6: molecular evolutionary genetics analysis version 6.0. *Mol. Biol. Evol.* 30 2725–2729. 10.1093/molbev/mst197 24132122 PMC3840312

[B40] Tatusova T. DiCuccio M. Badretdin A. Chetvernin V. Nawrocki E. P. Zaslavsky L. (2016). NCBI prokaryotic genome annotation pipeline. *Nucleic Acids Res.* 44 6614–6624. 10.1093/nar/gkw569 27342282 PMC5001611

[B41] Thorvaldsdóttir H. Robinson J. T. Mesirov J. P. (2013). Integrative genomics viewer (IGV): high-performance genomics data visualization and exploration. *Brief. Bioinform.* 14 178–192. 10.1093/bib/bbs017 22517427 PMC3603213

[B42] Wang Y.-Q. Zhang J.-S. Zhou J.-T. Zhang Z.-P. (2009). Biodegradation of 4-aminobenzenesulfonate by a novel *Pannonibacter* sp. W1 isolated from activated sludge. *J. Hazard. Mater.* 169 1163–1167. 10.1016/j.jhazmat.2009.04.002 19423220

[B43] Willems A. BUsse J. Goor M. Pot B. Falsen E. Jantzen E. (1989). *Hydrogenophaga*, a new genus of hydrogen-oxidizing bacteria that includes *Hydrogenophaga flava* comb. nov. (formerly *Pseudomonas flava*), *Hydrogenophaga palleronii* (formerly *Pseudomonas palleronii*), *Hydrogenophaga pseudoflava* (formerly *Pseudomonas pseudoflava* and “*Pseudomonas carboxydoflava*”), and *Hydrogenophaga taeniospiralis* (formerly *Pseudomonas taeniospiralis*). *Int. J. Syst. Evol. Microbiol.* 39 319–333. 10.1099/00207713-39-3-319

[B44] Yang Y. Q. Wu C. Wang L. Y. Yang L. (2012). Isolation and characterization of a sulfanilic acid degrading bacterial strain. *Appl. Mech. Mater.* 148–149, 46–49.

[B45] Yoon K. S. Sakai Y. Tsukada N. Fujisawa K. Nishihara H. (2009). Purification and biochemical characterization of a membrane-bound [NiFe]-hydrogenase from a hydrogen-oxidizing, lithotrophic bacterium, *Hydrogenophaga* sp. AH-24. *FEMS Microbiol. Lett.* 290 114–120. 10.1111/j.1574-6968.2008.01417.x 19025569

[B46] Yoon K.-S. Tsukada N. Sakai Y. Ishii M. Igarashi Y. Nishihara H. (2008). Isolation and characterization of a new facultatively autotrophic hydrogen-oxidizing Betaproteobacterium, *Hydrogenophaga* sp. AH-24. *FEMS Microbiol. Lett.* 278 94–100. 10.1111/j.1574-6968.2007.00983.x 18031533

