# Supplementary figures and images for: Nanopore Long-Read Guided Complete Genome Assembly of Hydrogenophaga intermedia, and Genomic Insights into 4-Aminobenzenesulfonate, p-Aminobenzoic Acid and Hydrogen Metabolism in the Genus Hydrogenophaga
Source: Front Microbiol. 2017 Oct 4;8:1880. doi: 10.3389/fmicb.2017.01880 (PMC5632844; doi:10.3389/fmicb.2017.01880)

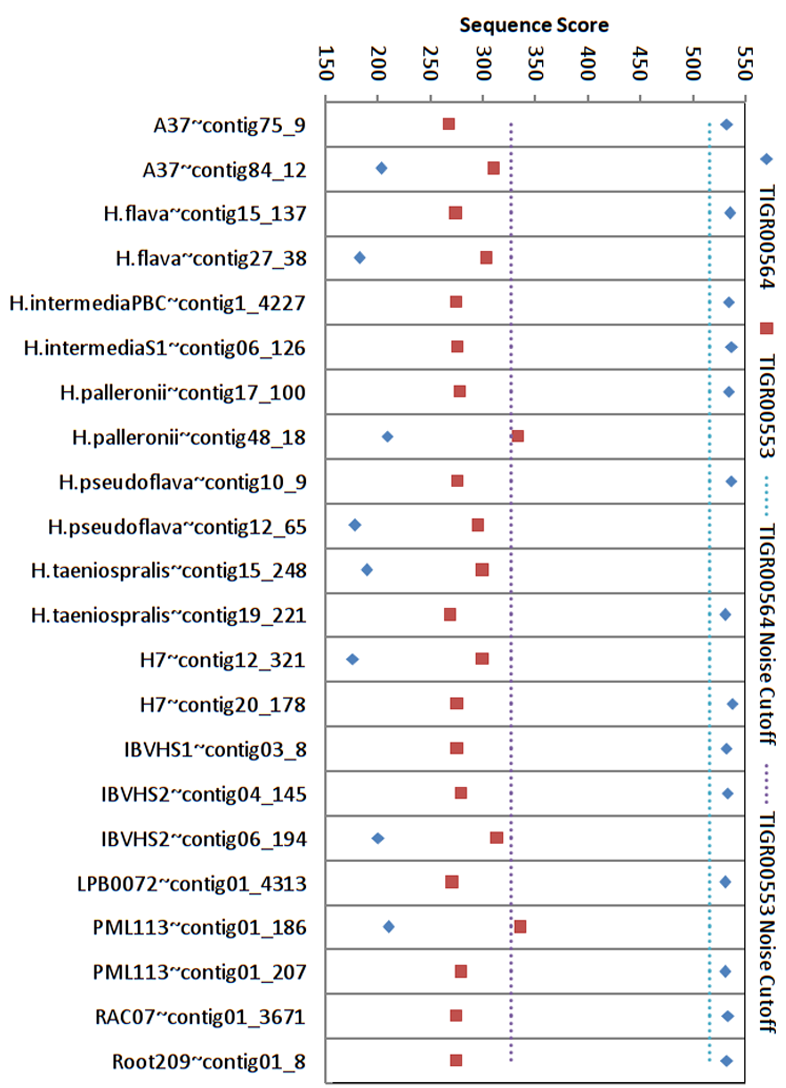

Supplement: Supplementary file 1 [file Image_1.TIF]

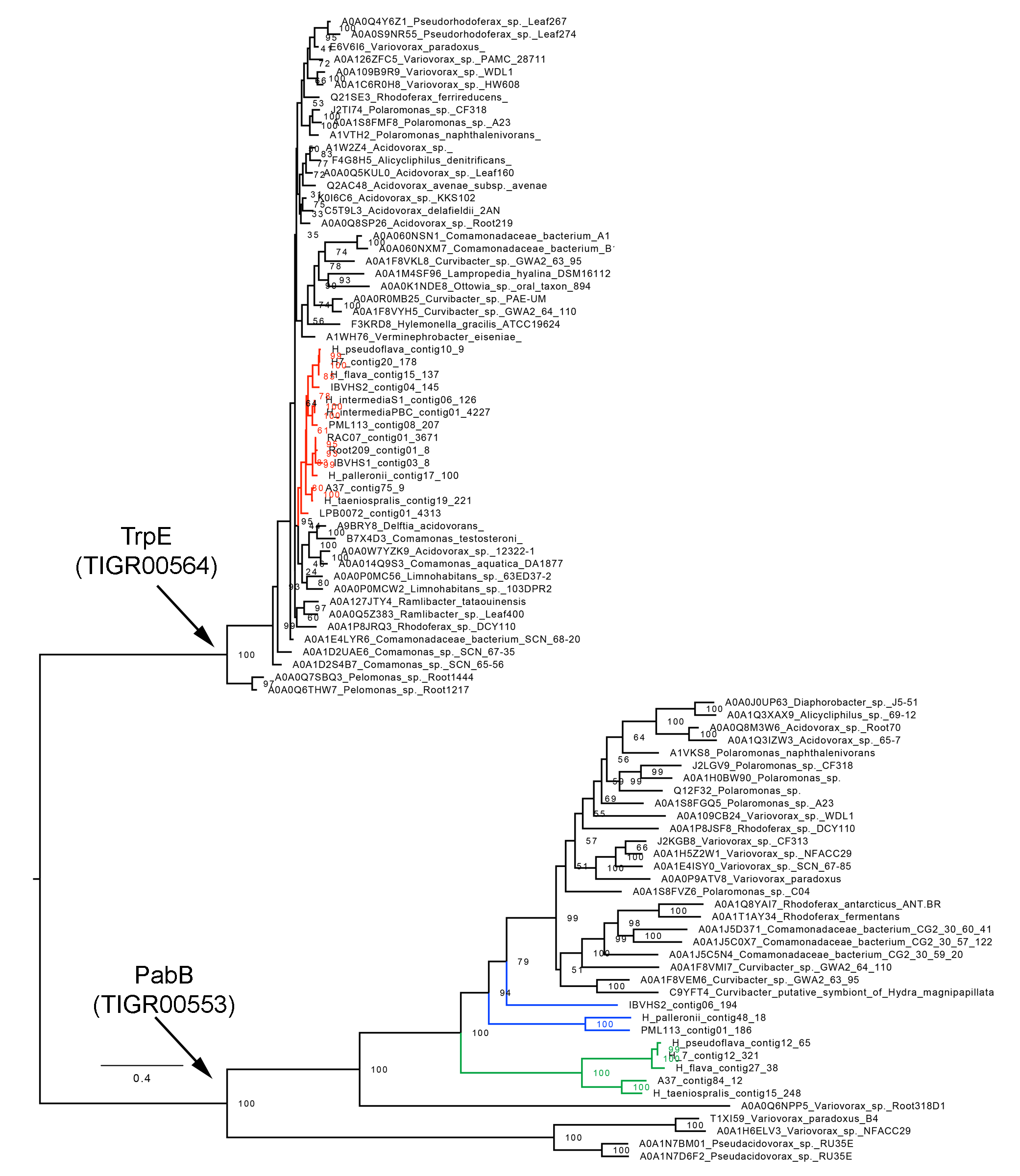

Supplement: Supplementary file 2 [file Image_2.TIF]
